# Assessment of changes in environmental factors in a tourism-oriented Island

**DOI:** 10.3389/fpubh.2022.1090497

**Published:** 2023-01-09

**Authors:** Zhipeng Shi, Yipeng Jiang, Xiaotong Zhai, Yuqing Zhang, Xiangming Xiao, Jianhong Xia

**Affiliations:** ^1^Human Settlements Research Center, Liaoning Normal University, Dalian, China; ^2^School of Marine Law and Humanities, Dalian Ocean University, Dalian, China; ^3^School of International Business, Liaoning Normal University, Dalian, China; ^4^Department of Microbiology and Plant Biology, Center for Earth Observation and Modeling, University of Oklahoma, Norman, OK, United States; ^5^School of Earth and Planetary Sciences (EPS), Curtin University, Perth, WA, Australia

**Keywords:** tourism urbanization, land cover transformation, land surface temperature, landscape index, Dachangshan Island

## Abstract

Tourism development has influenced industrial structure changes and has become a major driving force for China's new urbanization. However, the development will negatively impact natural resources and the ecological environment and will become an essential driving factor for land use change. Therefore, understanding the impact of tourism urbanization is crucial for sustainable local development. This study selected the Dachangshan Island in the Changhai County, Dalian, China, as the study area, because it is the only coastal island-type border county in China. During the study period, changes in local environmental factors were analyzed based on land use data, Landsat 5 and Landsat 8 data of 2009, 2014, and 2019. The results showed that: (1) the overall land surface temperature (LST) in the research region shows an increasing trend; the LST in 2014 and 2019 increased by 6.10 and 5.94 °C, respectively, compared with 2009. With respect to specific land types, impervious surfaces maintained a high land surface temperature (25.44, 32.38, and 31.86); however, surface temperatures for cropland, forest, grassland, and water bodies remained stable. (2) The land use land cover (LULC) change analysis from 2009–2019 indicates that impervious surfaces and cropland increased by 0.5653 km^2^ and 0.9941 km^2^, while the areas of forest, grassland, and water bodies decreased. The results also showed that forests (−1.3703 km^2^) are most affected by urbanization. (3) The results of the landscape index calculation showed that the variation at the patch scale is different for different LULC types. The patch density of impervious surfaces decreased, but the aggregation index increased over time, while the patch density of the forest increased continuously. At the landscape scale, overall patch type and distribution remained stable. The purpose of this study is to explore the environmental changes of islands and provide a reference for the sustainable development of islands.

## 1. Introduction

According to the National Bureau of Statistics, China's urbanization rate increased from 36.2 in 2000 to 60.6% in 2019. With the acceleration of urbanization, tourism has also seen rapid development, with statistics from the Ministry of Culture and Tourism in 2019 showing that tourism accounted for 11.05 percent of Gross Domestic Product (GDP). It can be seen that the tourism is an integral part of economic development. Previous studies show that the tourism industry is an essential driver of urbanization and that cities are the basis for developing the tourism industry; these two impact and constrain each other (1). It is therefore imperative that the role of tourism is brought into the context of sustainable development and that urbanization and tourism are integrated to promote co-development.

The tourism boom has not only contributed to the transformation of the local industrial structure, but also to the morphology and social evolution of rural communities, thus contributing to local economic development (2–5). Mullins first proposed the concept of tocapurism urbanization in 1991 (6). However, the process of tourism urbanization tourism is usually accompanied by an influx of tourists and an increase in traffic pressure, which affects residents' quality of life in tourist destinations (7–9). Adedoyin and Bekun (10) showed that tourism had become a more significant source of pollution than construction, further contributing to global carbon emissions. Recently, creating perfect urban facilities and services that guarantee smooth tourism activities has gathered increased attention, and an excellent tourism environment can enhance tourists' satisfaction and loyalty to the destination (11–13). However, rapid urbanization has also caused noticeable negative impacts, such as reduction of marine biodiversity, increased urban heat island effect, destruction of the ecological environment, and a decline in air quality (14–18). Furthermore, some studies have shown that the efficacy of tourism urbanization is affected by spatial planning (19). Therefore, when formulating relevant developmental policies, local governments should pay attention to balancing tourism and urbanization as much as possible while maintaining good economic development.

Recent studies on tourism urbanization are usually combined with air quality change, land use transfer, ecological, and environmental damage, and carbon emissions (20–23). These studies showed that factors such as the tourism industry, urbanization, ecological environment, and carbon emissions are mainly quantified, and a coupling analysis of these factors is carried out to analyze their evolution and spatial differences (10, 24, 25). For example, Li et al. (26) constructed a multi-indicator system to study the relationship between tourism urbanization and ecological and environmental elements in Chongqing, and the results show that the degree of coordination between the three increases over time. From the perspective of the research scale, it is mainly carried out from regions, urban agglomerations and tourism city scales (27–29), Gan et al. (30) used the gravity model of tourism economy to study the spatial characteristics of tourism economy in urban agglomeration in the middle reaches of the Yangtze River, in order to promote the cooperation and spatial integration of tourism economy in urban agglomeration. Foreign studies are mainly concentrated in tourism-oriented countries, while domestic studies are mainly distributed in eastern provinces and developed urban areas. However, correlation studies usually use longer time series of correlation data to analyze the interaction between factors.

The sudden outbreak of COVID-19 dealt a severe blow to tourism worldwide (31–33). Research on Spanish tourism by academics such as Arbulu showed that domestic travel dropped by 42.64% during the pandemic compared to 2019 (34). To rebuild and meet the needs of tourism and related development post-COVID-19 pandemic, land types often change and lead to changes in the local thermal environment (35–37). Among them, the most intuitive is an increase in impervious surfaces. Cities can divide the urban functional areas and relocate relevant industries to specific areas to improve the urban environment. However, the development of coastal cities is limited by land use to a certain extent (38). In addition, high temperatures and sea-level rise caused by high temperatures are obstacles to enhancing the economic potential of coastal tourist cities. Studies have shown that tourism-induced pollution and land-use change can also lead to changes in the carbon cycle and carbon sequestration capacity of vegetation, thereby exacerbating the impacts of climate change (15, 23). Recent studies have shown that climate change increase the vulnerability index of islands to climate change in developing countries (39–41). Therefore, it is imperative to analyze the changes in environmental factors in coastal tourism cities and islands.

Against the background of continuously practicing the strategy of building maritime power (a strategy proposed by the 18th National Congress of the Communist Party of China), islands have attracted many tourists with their unique natural conditions and fishing customs, which has promoted island tourism development (42–44). However, unlike land tourism cities, their unique geographical location and limited area have hindered such development to a certain extent. Cyffka et al. (45) found in their study, on the Elba Island and Malta, that the more tourism grew, the more serious the urban sprawl became, leading to the loss of the rural population. Hence, the interaction between tourism and urbanization will unavoidably cause alterations in the environmental elements of the islands. Therefore, the study of the changes in environmental factors on islands is not only conducive to environmental protection but also to the sustainable development of islands (46).

In summary, this study focused mainly on analyzing the changes in island environmental factors under tourism urbanization, including land surface temperature (LST), land cover, and landscape index-related factors. We chose the Changhai County, Dalian City, Liaoning Province, China, as the research area, which has formed a tourism industry with resource characteristics and folk characteristics based on natural and human resources. Therefore, the research contents of this paper mainly include three aspects: (1) analyzing the land surface temperature changes of islands during the period (2009–2019); (2) analyzing the land use changes during this period; and (3) using the land use data to calculate and analyze the corresponding landscape index and pattern change in the study area. We then provide suggestions for sustainable island tourism and island development.

## 2. Data and methods

### 2.1. Study area

**Figure 1:**
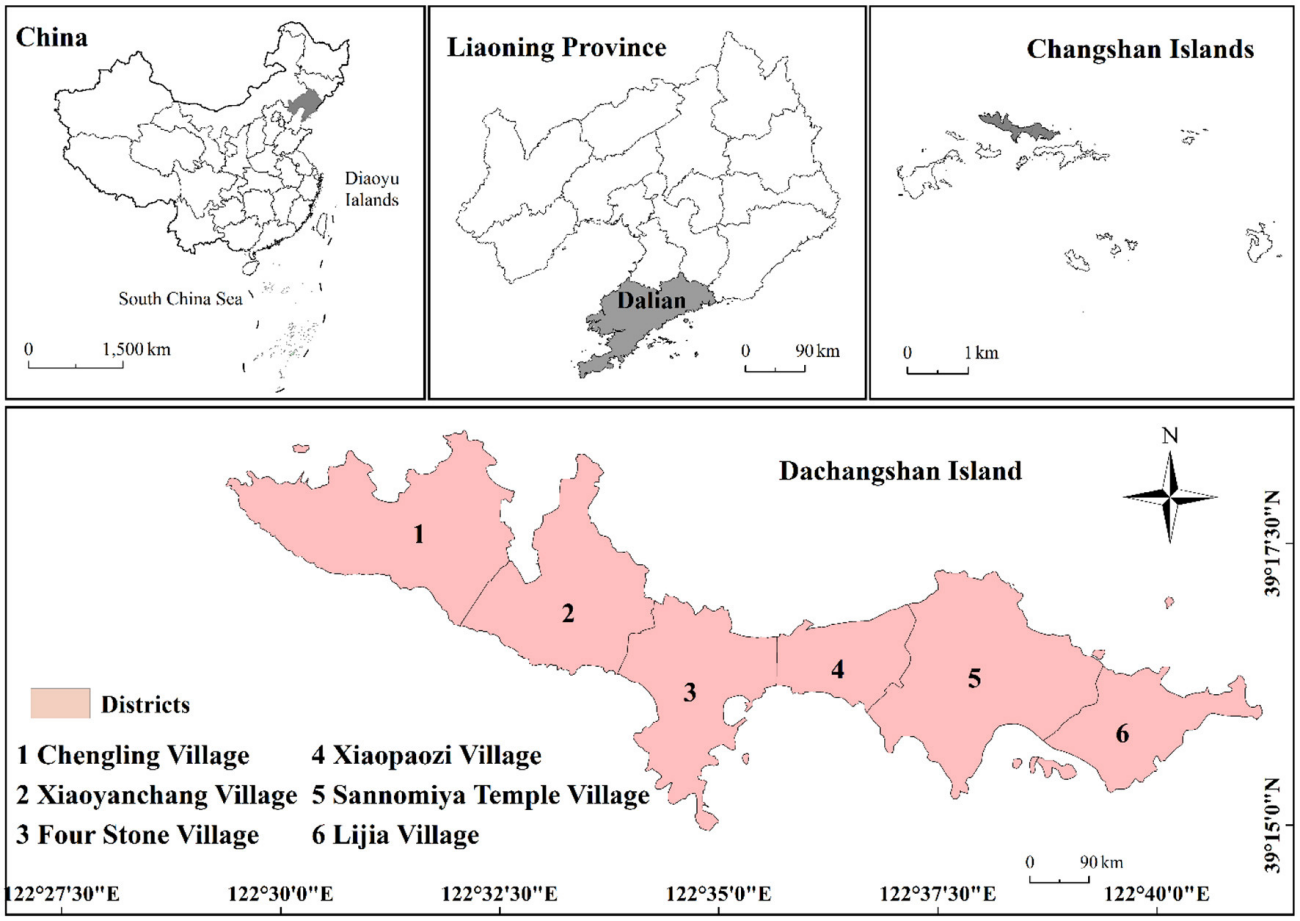
Location of the study area.

Dachangshan Island (122°57′ E, 39°27′ N) is located in the southeast of the Liaodong Peninsula and north of the Changshan Archipelago, and it's part of the Changhai County. The island is narrow from east to west and has a warm, temperate, and semi-humid climate characterized by four distinct seasons. The study area mainly includes the Lijia, Xiaopaozi, Sannomiya Temple, Xiaoyanchang, Chengling, and Four Stone Villages (Figure 1).

### 2.2. Data sources

**Figure 2:**
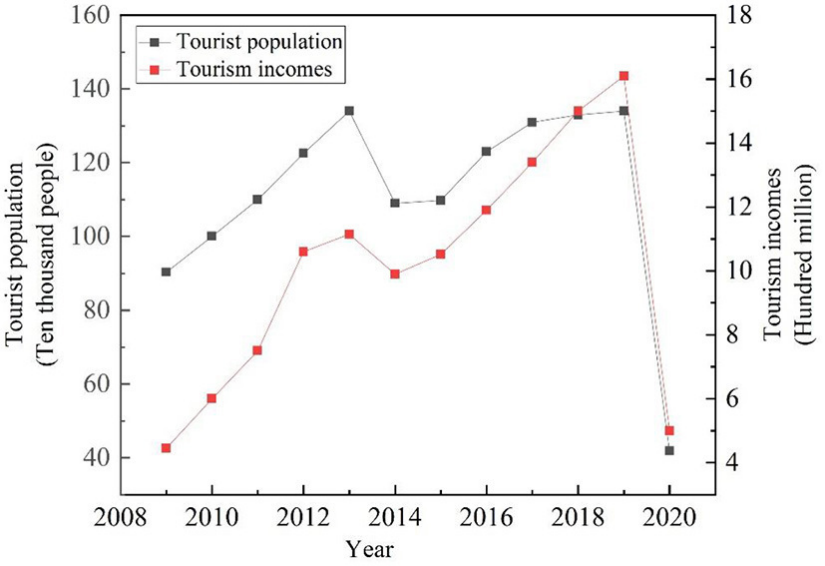
Tourism incomes and tourism population, 2009–2020.

This study analyzed tourism income and population data from the statistical bulletin of the Changhai County from 2009–2020 (Figure 2). Overall, tourism income and population showed an increasing trend year by year. In 2020, owing to the impact of COVID-19, the number of tourists and tourism income showed a sharp decline. The tourist population and income declined in 2014 but picked up afterwards. China Tourism Statistics Bulletin, suggests that overseas tourism was a potential factor that may have led to the decrease in tourist numbers and income in the study area. Meanwhile, 2020 was excluded owing to the impact of COVID-19. The purpose of this paper is to analyse the changes in environmental factors on the island during the summer months and, combined with the availability of remote sensing image data, we have finally chosen the land surface temperature and land cover data from 2009, 2014, and 2019, (Details of the data are shown in Table 1), and calculated the landscape index of each period.

**Table 1:** Data types and sources.

**Data types**	**Time**	**Resolution**	**Sources**
Landsat-5	2009.7.11	30 m	https://search.earthdata.nasa.gov/search/
Landsat-8	2014.7.6, 2019.7.4	30 m	http://www.gscloud.cn/
Land use data	2009, 2014, 2019	30 m	https://doi.org/10.5281/zenodo.4417810
Statistical yearbook data	2009-2020		http://www.dlch.gov.cn

### 2.3. Methods

#### 2.3.1. Land surface temperature inversion

LST inversion methods include the single-window algorithm, single-channel algorithm, radiative transfer equation algorithm and so on. Among which, the radiative transfer equation method has high inversion accuracy owing to a large number of input parameters and has been most widely used by scholars (44, 45). The mono-window algorithm is also widely used owing to its simplicity and universality. The single-window algorithm was adopted for this study because it has the strongest universality to retrieve the land surface temperature (47, 48). This method can convert the image DN value into the corresponding radiation intensity and then radiation brightness. The equations used are as follows:

(2.1) $$\begin{array}{l} L_{\lambda }\;=\;Gain\;\times \;DN\;+\;Offset \end{array}$$

(2.2) $$\begin{array}{l} T_{a}=\;\frac{K_{2}}{\ln\left(1\;+\;\frac{K_{1}}{L_{\lambda }}\right)} \end{array}$$

The inversion equation of land surface temperature is as follows:

(2.3) $$\begin{array}{l} T\text{\_}s=(a\left(1-C-D\right)+\left(b\left(1-C-D\right)+C+D\right)\\ T\text{\_}10-DT\text{\_}a)/\left(C-273.15\right) \end{array}$$

(2.4) $$\begin{array}{l} C=\varepsilon \tau \end{array}$$

(2.5) $$\begin{array}{l} D=\left(1\;-\tau \right)\left[1\;+\;\left(1\;-\varepsilon \right)\tau \right] \end{array}$$

In Equation (2.1), L_λ_ is the radiation intensity, Gain is the gain factor, and Offset is the offset function (both of which can be obtained from image metadata). According to the data sources K_1_ and K_2_, Equation (2.2) the different data correspond to different parameters. In Equation (2.3), T_s is the surface temperature value (K), where T_a is the average temperature of the atmosphere (K), T_10 is the luminance temperature (K) of the sensor, a and b are reference coefficients (when the surface temperature is between 0 and −70 °C, a = −67.355351, b = 0.458606), τ is the propagation of the atmosphere, and ε is the surface emissivity.

#### 2.3.2. Land use data

To understand land cover change in the research region, we used the land cover data generated by Yang et al. (49) based on the Google Earth Engine (GEE), which covers the land use dynamics in China from 1990–2019. It is divided into ten land cover types, and the verification results show that the overall accuracy is as high as 79.31%. In this article, the land cover data for 2009, 2014, and 2019 were extracted, and the land transfer matrix was drawn for the land change in these two periods to analyze the land cover change of islands in different periods.

#### 2.3.3. Calculation of landscape index

The landscape index can indicate information about the landscape pattern of land use types in a specific region. The scale of the study can be classified into patch level indices, patch type level indices and landscape level indices (50). The study area was examined from the patch-type and landscape levels for this study. Finally, we selected the patch density, aggregation index, landscape shape index, and Shannon diversity index to analyze the landscape changes of the Dachangshan Island. Table 2 presents the meaning of each index and the calculation method. The entire process was performed using Fragstats v4.2.1.

**Table 2:** Calculation method and description of landscape index.

**Landscape index**	**Equation**	**Unit and value range**	**Explain**
Patch density	*PD* = (10000)(100)	Num/100 ha (0,+∞)	The larger the number of plaques per unit area, the finer the segmentation of plaques
Aggregation index	$AI=\left[\frac{g_{ii}}{max\to g_{ii}}\right]\left(100\right)$	Percent (0,100]	Degree of patch aggregation in landscape
Landscape shape index	$LSI=\frac{.25\;E^{*}}{\sqrt{A}}$	None [1, +∞)	Describe the characteristics of patch shape in the whole landscape, the larger the value, the more isolated the patch
Shannon's diversity index	$SHDI=-\overset{m}{\underset{i=1}{\sum }}\left(P_{i}\times InP_{i}\right)$	[0,100)	The larger the value, the more abundant the patch types and distribution in the landscape

## 3. Results

### 3.1. Land surface temperature change

**Figure 3:**
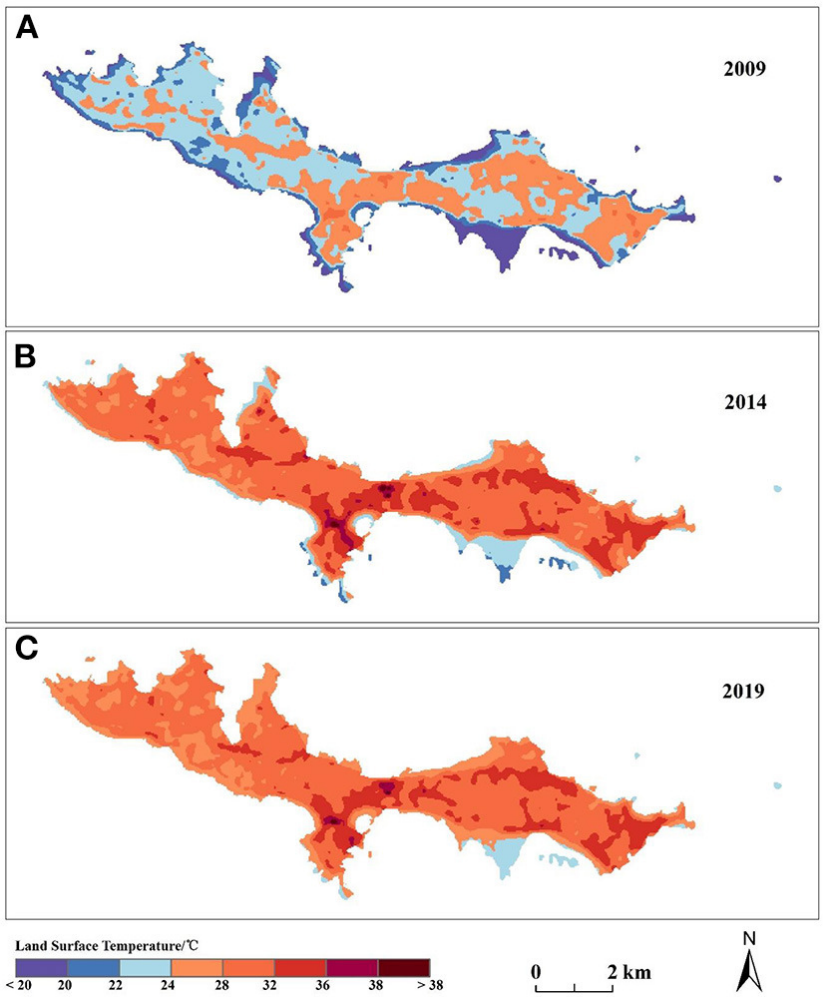
**(A–C)** Show the land surface temperature in 2009, 2014, and 2019, respectively.

**Figure 4:**
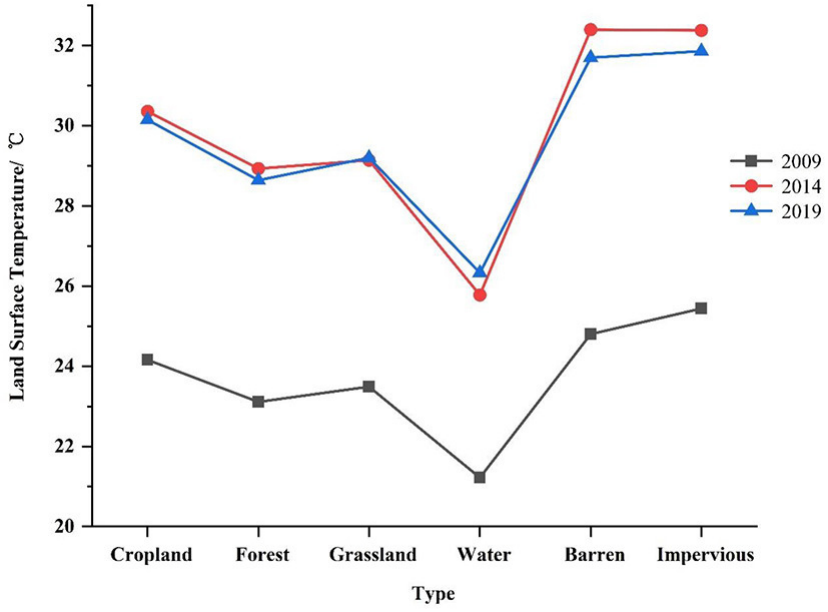
Land mean surface temperature changes in 2009, 2014, and 2019 by local table types.

**Figure 5:**
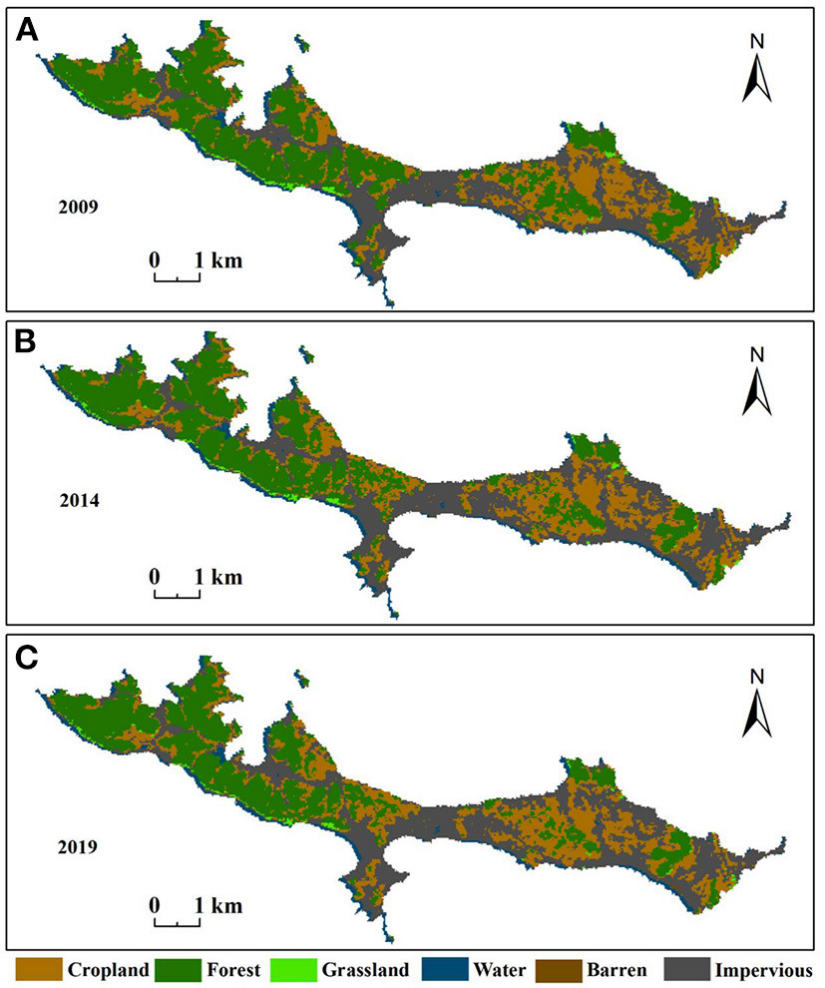
**(A–C)** Shows the different land use in 2009, 2014, and 2019, respectively.

**Figure 6:**
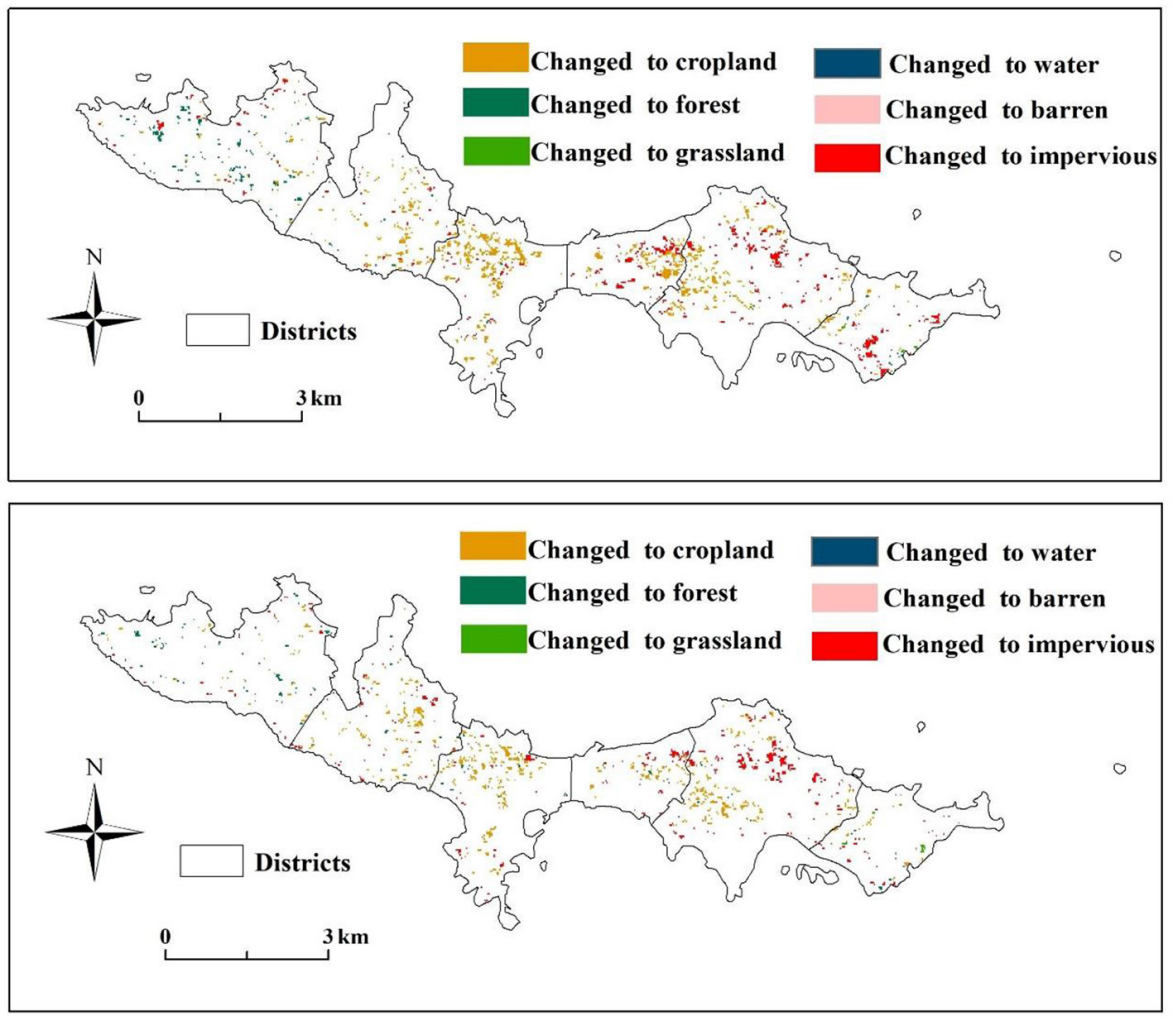
Land use transfer and change from 2009–2014 and from 2014–2019.

The LST is a critical indicator that reflects the urban thermal environment. Figure 3 shows the land surface temperature distribution on the Dachangshan Island in 2009, 2014, and 2019, respectively. As only one meteorological station exists in the study area, we chose to use ASTER__08 LST data for accuracy validation, and due to data quality limitations, we only validated the temperature accuracy for 2014 as well as 2019. We extracted the corresponding temperature values separately by creating random points in Arcgis 10.5 software and performed regression analysis on both. The results showed that the inverse surface temperature Pearson correlation coefficients were 0.816 and 0.808 for 2014 and 2019. Where the surface temperature increased by 6.10°C and 5.94°C in 2014 and 2019 respectively compared to 2009. The high-temperature areas were mostly located in the central and eastern parts of the island, while the western part usually had low temperatures. Figure 5 shows that the central and eastern parts of the island consist mainly of impervious areas. Also, in conjunction with Figure 6, it was found that the increase or decrease in impervious surface was mainly concentrated in the eastern part of the island during the two time periods 2009–2014 and 2014–2019. Vegetation was mainly found in the western part of the island, and the 2019 statistical bulletin of Changhai County indicates that the local forest cover reached 47.78% at the end of the year. In the context of global warming, there are also differences in surface temperature between different land covers, so this paper also analyses the changes in surface temperature over three time periods under different land cover types (Figure 4); the results show that:the average land surface temperature of each land type during the study period generally showed an increasing trend. For instance, barren and impervious surfaces show higher LST values than of the others in any period, with the highest variation and continuous increase in the range from 2009–2014. The higher LST values for these two surfaces can be attributed to the nature of their surface cover and characteristics (low specific heat capacities, and high reflectivity) (51). However, although cropland, forest, grassland, and water also showed significant increases, the overall values were lower than those of the barren and impervious categories. This is because vegetation can usually alleviate the temperature rise through transpiration, whereas water has a higher specific heat capacity, so it usually has a lower temperature (49, 50).

### 3.2. Land use change

To reflect land cover change in the study area, land use maps were created for each period (Figure 5). In general, the land cover of the research area had minor changes. The western part was still dominated by forest, while the central and eastern parts were dominated by mainly impervious, cropland, and other land types. The land use transfers during the study are presented in Figure 6. Significant increased in cropland and impervious surfaces during this period. Cropland increased throughout the island, whereas the changes to impervious surfaces was predominantly concentrated in the eastern part of the island. The land use change from 2009 to 2014 is shown in Table 3; cropland and impervious areas increased by 0.3511 km^2^ and 0.5673 km^2^, respectively. The areas of barren forest, grassland, and water were reduced, with forest being the most affected (0.8378 km^2^). From 2014–2019 (Table 4), cropland (0.2141 km^2^) and impervious (0.4269 km^2^) areas increased, while forest (0.5326 km^2^), grassland (0.0382 km^2^), and water (0.0702 km^2^) decreased. Combined with Figure 1, it was found that impervious area increased mainly in Xiaobaozi Village, Sanguangmiao Village and Lijia Village from 2009-2014, while impervious area increased mainly in Sanguangmiao Village from 2014-2019, while the increase in cultivated land was mainly concentrated in Sishi Village, but it tended to be surrounded by large areas of forest land and was therefore more dispersed.

**Table 3:** Land use change from 2009–2014 (km^2^).

**Land cover 2014**								
**Land cover**		**Barren**	**Cropland**	**Forest**	**Grassland**	**Impervious**	**Water**	**Sum**
2009	Barren	0.0146	0	0	0	0.0049	0	0.0195
	Cropland	0	5.1168	0.1460	0.0188	0.4568	0	5.7384
	Forest	0	0.9295	8.7053	0	0.0688	0	9.7036
	Grassland	0.0014	0.0389	0.0146	0.2537	0.0146	0	0.3233
	Impervious	0	0	0	0	7.6025	0.0049	7.6074
	Water	0	0.0042	0	0	0.0271	1.2333	1.2646
	Sum	0.0160	6.0895	8.8659	0.2725	8.1747	1.2382	24.6567
	Change	−0.0035	0.3511	−0.8378	−0.0507	0.5673	−0.0264	

**Table 4:** Land use change from 2014–2019 (km^2^).

**Land cover 2019**								
	**Land cover**	**Barren**	**Cropland**	**Forest**	**Grassland**	**Impervious**	**Water**	**Sum**
2014	Barren	0.0146	0	0	0	0.0014	0	0.0160
	Cropland	0	5.6571	0.1098	0.0195	0.3031	0	6.0895
	Forest	0	0.6132	8.2151	0.0028	0.0348	0	8.8659
	Grassland	0.0014	0.0306	0.0063	0.2120	0.0222	0	0.2725
	Impervious	0	0	0	0	8.1726	0.0021	8.1747
	Water	0	0.0028	0.0021	0	0.0674	1.1659	1.2382
	Sum	0.0160	6.3036	8.3333	0.2343	8.6016	1.1679	24.6567
	Change	0.0000	0.2141	−0.5326	−0.0382	0.4269	−0.0702	

Lijia, Xiaopaozi, Sannomiya Temple, Xiaoyanchang, Chengling, and Four Stone Villages.

### 3.3. Landscape index results

The landscape index was used to analyze the landscape characteristics in 2009, 2014, and 2019, and and the results of the calculations are shown in Table 5. The land use change analysis results using the patch type scale for 2009–2019 indicate that cropland, grassland, and impervious type patch density exhibited decreasing trends, demonstrating that patch attributes gradually tend to be homogeneous in per unit area. In contrast, the aggregation index of impervious increased during the same period. The patch density of water and forest showed a gradual increase, indicating that the fragmentation degree of these two landscapes continuously increased. The fragmentation of the forest is mainly evident in Four Stone Village, where forest and corpland are interspersed. However, the change in the aggregation index of water was greater than that of the forest, and the aggregation index of water was as high as 85.0641 in 2019. In addition, the barren patch density and aggregation index reached their highest levels in 2014. From the overall landscape scale, it was found that the landscape shape index first increased and then decreased from 2009–2019, with 2014 as the cut-off point, indicating that the patches in the whole study area had experienced a transition from separation to integration. With the development of the island, different functional land uses gradually concentrated with each other to rationalize the use of resources and improve land use efficiency. However, the Shannon diversity index was stable (0.9195) in 2009 and 2014, but slightly decreased (0.9180) in 2019. This result suggests that the patch types and distribution in the study area did not change significantly during this period.

**Table 5:** Landscape index calculation for 2009, 2014, and 2019.

	**2009**		**2014**		**2019**	
	**PD**	**AI**	**PD**	**AI**	**PD**	**AI**
Cropland	1.9094	72.9598	1.8173	72.6306	1.5336	74.8115
Forest	0.4524	89.9972	0.6288	88.3603	0.6595	88.7919
Grassland	0.7131	41.1765	0.6441	42.0593	0.5828	38.2143
Water	0.4754	65.6355	0.4831	65.4198	0.5138	85.0641
Barren	0.0613	31.2500	0.0383	37.0370	0.0537	33.3333
Impervious	1.3266	81.9858	1.2346	82.9733	1.1962	83.7299
LSI	10.5997		10.8255		10.5453	
SHDI	0.9195		0.9195		0.9180	

## 4. Discussion

To meet the development of tourism, the land use and land cover of islands have changed because of anthropogenic factors. In 2014, the website of the Central People's Government of the PRC issued several opinions on promoting the reform and development of tourism, pointing out that tourism is an essential component of the modern service industry, and accelerating the reform and development of tourism is an inevitable requirement to adapt to the upgrade in people's consumption and the industry's structure. Moreover, according to the National Bureau of Tourism Statistics, the number of outbound tourists exceeded 100 million for the first time in 2014, which is also a potential factor that may have caused decrease in visitor numbers and revenue in 2014. However, the decrease in the number of tourists creates a peculiar opportunity for the restoration of the local ecological environment. Previous studies on tourism have used statistical data to analyse the impact of tourism on the local economy, population and other aspects, while statistical data is often only available until the relevant agencies can obtain it at a specific time, this paper uses remote sensing data to evaluate the environmental factors of the island in a number of ways that are more timely and objective than statistical data. Table 6 shows that from 2009–2019, the impervious area increased the most (0.9941 km^2^), followed by cropland (0.5653 km^2^). Barren land, forest, grassland, and water tended to decrease. Among them, forests decreased the most (−1.3703 km^2^), and an analysis of land use change shows that forests are the most vulnerable land cover type on islands of limited size compared to other land covers. Especially in Four Stone Village, where Cropland and Forest are interspersed, it is important to meet the sustainable development of the island Corpland while protecting the ecological environment. In addition, related studies have shown that vegetation can cool cities through transpiration and provide shade (51, 52). Furthermore, other studies have found that the configuration of green spaces has different effects on temperature mitigation (53–55), implying that for islands with minimal land area, more focus should be given to rational planning of local land types while promoting tourism development.

**Table 6:** Land use change from 2009–2019 (km^2^).

**Land cover 2019**								
**Land cover**		**Barren**	**Cropland**	**Forest**	**Grassland**	**Impervious**	**Water**	**Sum**
2009	Barren	0.0132	0	0	0	0.0063	0	0.0195
	Cropland	0	4.7588	0.2044	0.0271	0.7481	0	5.7384
	Forest	0	1.4753	8.1074	0.0063	0.1147	0	9.7036
	Grassland	0.0028	0.0626	0.0195	0.2009	0.0375	0	0.3233
	Impervious	0	0	0	0	7.6025	0.0049	7.6074
	Water	0	0.0070	0.0021	0	0.0925	1.1631	1.2646
	Sum	0.0160	6.3036	8.3333	0.2343	8.6016	1.1679	24.6567
	Change	−0.0035	0.5653	−1.3703	−0.0889	0.9941	−0.1015	

The change of land cover and the difference in land properties led to the corresponding change in LST. When Ara et al. (56) analyzed the correlation between the tourist pressure index and land use and surface temperature, they found that with an increase in the tourist pressure index, land use changes became more frequent, further aggravating surface temperature changes. Under the background of global warming, the island's overall land surface temperature is rising, the Impervious and Barren most obvious (Figure 4), with a simultaneous temperature rise in Cropland, Forest, Grassland, and Water. Safarrad et al. (57) analyzed the impact of tourism on LST and found that the LST between tourist areas and other regions was significantly different. In addition to the changes in natural elements, tourism is usually accompanied by a large inflow of people into the designated islands, during which, a large amount of anthropogenic heat will also be generated, resulting in heat discomfort, which will negatively impact the physiology and psychology of tourists (28, 58). Whereas, tourists usually stay in tourist areas for a specific period of time, the state of the environment is critical to the sustainability of the local area for those living on the island for long periods of time, and it is shown in chapter 3.2 that forests are the most vulnerable surface type, and that forests have a significant effect on cooling. This requires the local government to plan the island's land use in a way that meets the needs of tourism development and maintains the balance of the island's environment in the process of developing tourism.

The related landscape pattern indices can quantitatively reflect landscape characteristics (59). However, it must be screened according to the actual situation. The results indicated that the landscape diversity in the research area was stable, and the landscape types were gradually integrated. In addition, some scholars have divided the research area into tourism and non-tourism land based on the data of interest, analyzed the changes in LST, normalized index, and land use between them, and provided suggestions for sustainable development of local tourism (60, 61). However, this method is suitable mainly for tourism in cities, where, unlike on islands with limited land areas, life and tourism intersect, making it difficult to define tourism and non-tourism.

### 4.1. Limitations

This study analyzed the impact of tourism on the environmental factors of the Dachangshan Island from 2009–2019. However, due to the limitation of image quality and time, remote sensing data of the same day could not be obtained; thus, there were certain deficiencies in the analysis of land surface temperature changes. In addition, because of the limited study area, we selected land use data with a resolution of 30 m to ensure data consistency. There may be an incomplete representation of the actual land cover in the study area. Therefore, higher-resolution data should be used for analysis when studying islands with limited areas.

## 5. Conclusions

To better understand the changes in the environmental factors of tourism-oriented islands, this study used multi-source data to analyze the environmental factors of the Dachangshan Island based on tourism income and the number of tourists. The key findings are as follows:

1. The land surface temperature in the study area exhibited a rising trend during the periods considered, with impervious and barren areas having a higher LST. The cropland, forest, grassland, and water surface temperatures increased evenly; however, they were lower than those in impervious and barren areas.
2. The western part of the research area is still mainly composed of forests, whereas the central and eastern parts of the research area are composed of impervious, cropland, and other land types. Land use changes during 2009–2019 showed that overall forest, grassland, water, and barren areas reduced, while cropland and impervious areas increased.
3. The calculation results of the landscape index showed that from 2009–2019, land types show different trends of change. At the patch type scale, the most noticeable impervious patch decreased continuously, while the aggregation index increased continuously, indicating that imperviousness gradually integrated with increasing anthropogenic factors.

## Data availability statement

The original contributions presented in the study are included in the article/supplementary material, further inquiries can be directed to the corresponding authors.

## Author contributions

ZS wrote the main manuscript text. YJ, XZ, YZ, XX, and JX conducted the experiment and analyzed the data. All authors reviewed the manuscript. All authors contributed to the article and approved the submitted version.
